# On Infanticide

**Published:** 1802-11-01

**Authors:** 


### *To the Editors of the Medical and Phyſical Journal.*

GENTLEMEN,

SEVERAL reflexions naturally aroſe in my mind, on peruſing Mr. Chamberlaine's paper in your Journal of laſt March. The circumſtance he alludes to, namely, the means of determining with preciſion, whether a child has been born alive or not, has ever appeared to me to involve conſequences of the greateſt moment, both to ſociety and to the individual. But does the certainty of the child having been born alive, always imply criminality in the mother? Certainly not. Or, are we invariably to infer guilt, becauſe a child is found at the bottom of a river, or ſmothered in a privy? Certainly not. In determining the guilt of the parent, every circumſtance, however trivial it may appear, ſhould be minutely and deliberately weighed. Neither ſhould we bring the unhappy mother to puniſhment, unleſs the circumſtances are ſo ſtrong as to remove all doubt from the minds of the moſt heſitating and timid. I have been pretty extenſively engaged in midwifery, and have repeatedly witneſſed the death of the child a few hours, nay, even minutes after its birth. Let us then imagine an unfortunate tunate woman placed under fuch circumftances.— By art, perhaps by falfehood, fhe has contrived hitherto to deceive the world; fhe has ftill fome character at ftake; fome parent whofe diftrefs fhe may be anxious to fpare. Stung with anxiety and remorfe, without any friend to advife, any counfellor to direct, fhe feizes her infant, which has juft expired, and without thought, without prudence, without judgment, fhe madly rufhes to the firft river, pond, privy, or dung-heap, and buries, as fhe fondly hopes, her infant and her fhame together.

I am, however, more particularly induced to addrefs this letter to you, from the following circumftances, which occurred to me a few years ago, and which would have been ftrongly in my recollection had I ever been called upon to give my opinion on fuch a fubject in a court of juftice.

Mrs. S. whom I had attended in former labours, was in a fubfequent pregnancy, towards the termination of her reckoning, attacked with diarrhœa, and had regularly gone to the common neceffary; but being one day above ftairs, the inclination to go to ftool was fo urgent, as to oblige her to ufe a night chair half full of water. No fooner, however, was fhe feated on it, than a fevere pain of labour came on, and continued without intermiffion till the child was born, and without the poffibility of her removing or alarming the family previous to the delivery. I was immediately fent for, and refcued the child, who is now living, from its perilous fituation, without its having received any injury. Had Mrs. S. been below ftairs, or in the garden, the confequences to the infant might have been fatal. But in *this* cafe, no poffible criminality could have attached to the mother, for *her character was above suspicion.* Place then an *unmarried* female in a fimilar fituation, and afk what would have been the general impreffion refpecting *her*, had fhe loft her child? Scarcely, alas! would this unhappy mother have found an advocate; the general voice would have been loud in her condemnation, and perhaps, with fatisfaction, have feen her led to punifhment. With compaffion, therefore, for the failings of others, let us, as men, not be too hafty in adopting an opinion, nor too tenacious in maintaining it; let us feel for the mifery; we who boaft not only the fuperior ftrength of our bodies, but alfo that of our minds—let us, I fay, feel for the mifery we are fo inftrumental in caufing, and fhudder at the poffibility of fteeling our hearts to the fufferings of a too credulous, perhaps of a too fond affociate in our guilt.

The theorift may conclude it very eafy to diftinguifh actual labour pains from the bearing down or inclination to ftool; but the man of practice will correct him, becaufe he daily finds it otherwife. Labour, alfo, in many females, advances with uncommon uncommon rapidity, and children are frequently born with scarcely more than one protracted pain. "A lady (in the paper alluded to, p. 284) was taken so suddenly in labour, *that the child shot forth* from her with such force as to separate the funis."

Warned by the case of Mrs. S. this rule in Midwifery should never be forgotten, that towards the end of pregnancy, when there is a probability of the child living if born, women should guard against all hazard of losing it, by avoiding a common privy; and this more especially when the bowels are tender, or the preceding labours have terminated quickly. I am, &c.

